# Perlecan: a review of its role in neurologic and musculoskeletal disease

**DOI:** 10.3389/fphys.2023.1189731

**Published:** 2023-05-30

**Authors:** Tessa R. Lavorgna, Timothy E. Gressett, Wesley H. Chastain, Gregory J. Bix

**Affiliations:** ^1^ Tulane University School of Medicine, New Orleans, LA, United States; ^2^ Clinical Neuroscience Research Center, Department of Neurosurgery, Tulane University School of Medicine, New Orleans, LA, United States; ^3^ Tulane School of Medicine, Tulane Brain Institute, New Orleans, LA, United States

**Keywords:** Schwartz-Jampel Syndrome, perlecan, neurologic disease, alzheheimer’s disease, ischemic stroke, musculoskeletal disorders, osteoarthiritis, sarcopenia

## Abstract

Perlecan is a 500 kDa proteoglycan residing in the extracellular matrix of endothelial basement membranes with five distinct protein domains and three heparan sulfate chains. The complex structure of perlecan and the interaction it has with its local environment accounts for its various cellular and tissue-related effects, to include cartilage, bone, neural and cardiac development, angiogenesis, and blood brain barrier stability. As perlecan is a key contributor to extracellular matrix health involved in many tissues and processes throughout the body, dysregulation of perlecan has the potential to contribute to various neurological and musculoskeletal diseases. Here we review key findings associated with perlecan dysregulation in the context of disease. This is a narrative review article examining perlecanâ€™s role in diseases of neural and musucloskeletal pathology and its potential as a therapeutic index. Literature searches were conducted on the PubMed database, and were focused on perlecan’s impact in neurological disease, to include ischemic stroke, Alzheimer’s Disease (AD) and brain arteriovenous malformation (BAVM), as well as musculoskeletal pathology, including Dyssegmental Dysplasia Silverman-Handmaker type (DDSH), Schwartz-Jampel syndrome (SJS), sarcopenia, and osteoarthritis (OA). PRISMA guidelines were utilized in the search and final selection of articles.Increased perlecan levels were associated with sarcopenia, OA, and BAVM, while decreased perlecan was associated with DDSH, and SJS. We also examined the therapeutic potential of perlecan signaling in ischemic stroke, AD, and osteoarthritic animal models. Perlecan experimentally improved outcomes in such models of ischemic stroke and AD, and we found that it may be a promising component of future therapeutics for such pathology. In treating the pathophysiology of sarcopenia, OA, and BAVM, inhibiting the effect of perlecan may be beneficial. As perlecan binds to both Î±-5 integrin and VEGFR2 receptors, tissue specific inhibitors of these proteins warrant further study. In addition, analysis of experimental data revealed promising insight into the potential uses of perlecan domain V as a broad treatment for ischemic stroke and AD. As these diseases have limited therapeutic options, further study into perlecan or its derivatives and its potential to be used as novel therapeutic for these and other diseases should be seriously considered.

## 1 Introduction

Perlecan is a 500 kDa proteoglycan parent molecule residing in the extracellular matrix of basement membranes with five distinct protein domains and three heparan sulfate chains at the N terminus (Roberts et al., 2012; Gubbiotti et al., 2017). The diversity of its structure lends itself to perlecan’s far reaching cellular effects, including cartilage, bone, neural and cardiac development (Roberts et al., 2012; Gubbiotti et al., 2017; Martinez et al., 2018), angiogenesis (Lee et al., 2011; Gubbiotti et al., 2017; Nakamura et al., 2019; Trout et al., 2021), blood brain barrier stability (Lee et al., 2011; Gubbiotti et al., 2017; Nakamura et al., 2019), atherosclerotic plaque formation (Gubbiotti et al., 2017; Trout et al., 2020), and possibly even as a modulator for Sars-CoV-2 binding and related pathophysiological effects (De Pasquale et al., 2021). Perlecan is derived from the *HSPG2* gene found on chromosome 1 (Dodge et al., 1995; Gubbiotti et al., 2017). Perlecan knock-out animal models are currently lacking as *Hspg2* ^−/−^ mice experience embryonic lethality with severe effects on neural, cardiac, and cartilaginous development (Arikawa-Hirasawa et al., 1999; Ishijima et al., 2012). While basement membranes initially form in perlecan-null mice, they are inherently unstable and unable to stand shear stress which leads to rapid deterioration, with death occurring from blood leakage into the pericardial cavity or respiratory failure from a developmentally dysplastic rib cage (Arikawa-Hirasawa et al., 1999; Costell et al., 1999). Therefore, perlecan is thought to be essential in stabilizing the extracellular matrix required for vital organ development and function.

Intriguingly, some mouse models which alter levels of perlecan expression mimic the clinical phenotype seen in severe chondrodysplasia disorders, including the embryonic lethal *HSPG2* knock-out disorder, DDSH, and the *HSPG2* knock-down disorder known as SJS. Perlecan has also been implicated in the pathophysiology of non-developmental genetic musculoskeletal diseases, such as sarcopenia and OA (Kaneko et al., 2013; Wang et al., 2014; Gubbiotti et al., 2017). The mechanism regulating such cellular effects has classically been described as perlecan interacting with VEGFA of the *VEGFR2* family, and by stimulating FGFR receptors to promote neovascularization (Aviezer et al., 1994; Zoeller et al., 2009; Gubbiotti et al., 2017). As diminished capillary health is a critical component in the pathophysiology of some neural diseases, there is potential for perlecan and its angiogenic C terminal domain V to serve as an effective translational therapeutic agent. In stroke models, for example, perlecan acts on the α5β1 integrin receptor and VEGFR2 and PDGFRβ receptors to increase VEGF production and angiogenesis (Parham et al., 2014; Nakamura et al., 2019; Trout et al., 2020). Furthermore, there is some evidence of perlecan diminishing the neurotoxic amyloid plaque buildup in diseases such as AD (Parham et al., 2014). Dysregulated angiogenesis can result in multiple pathologies including BAVM, and thus, perlecan’s role in angiogenesis may provide further insight into this disorder as well (Kim et al., 2009).

While current research describes perlecan in context of singular diseases, no comprehensive review exists which examines its involvement in common neural and musculoskeletal diseases. Here we review and summarize the role of perlecan in neural and musculoskeletal disease and examine its possible use as a novel therapeutic target and discuss its role in angiogenesis and overall health functions in relation to perlecan expression and clinical features.

## 2 Methods

This is a narrative review article examining perlecan’s role in diseases of neural and musculoskeletal pathology and its potential as a therapeutic index. Analysis was conducted by utilizing the key phrases “perlecan” AND “brain,” “perlecan” AND “cartilage” and “Schwartz-Jampel Syndrome” in the PubMed database. Between these three searches, 32 peer-reviewed journal articles were selected for relevancy, and all abstracts were reviewed. From this initial selection, 25 articles were read at full length, and 19 were deemed relevant and thus included in this narrative review. We focused on perlecan’s impact in neurological disease, including ischemic stroke, AD, and BAVM as well as musculoskeletal pathology, including DDSH, SJS, sarcopenia, and OA. PRISMA guidelines were utilized in the search and final selection of articles (Page et al., 2021).

## 3 Results

### 3.1 Perlecan animal models

**FIGURE 1:**
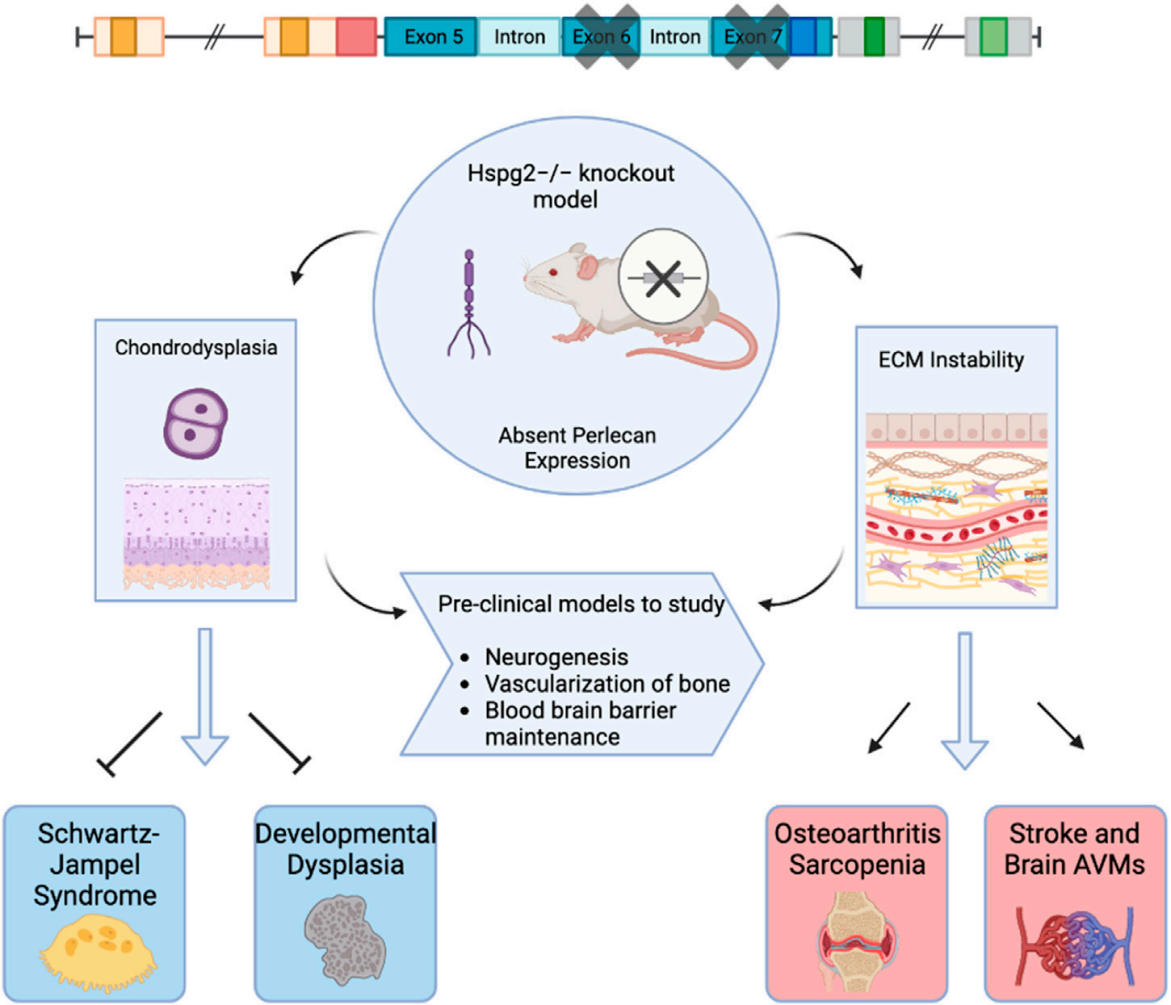
Graphical Abstract. This figure depicts perlecan knockout mice models used to examine biochemical effects of absent perlecan expression on mice embryos with effects on subsequent clinical pathology. Created with BioRender.com.

Various animal models have been used to study the effects of perlecan. In 1999, both Costell and Arikawa-Hirasawa et al. developed perlecan knockout mice models (Arikawa-Hirasawa et al., 1999; Costell et al., 1999). Costell et al. developed perlecan knockout (KO) mice (i.e., Hspg2^−/−^) by removing exon 6 of the perlecan gene (Costell et al., 1999). Such homozygous knockout models resulted in chondrodysplasia, cleft palate, microaneurysms, and hemopericardium. Further examination of these models posited that inability to withstand stress on developing cartilage and cardiac basement membranes led to respiratory failure and fatality soon after birth (Costell et al., 1999). Arikawa-Hirasawa et al. removed exon 7 of the *HSPG2* gene, resulting in homozygous mutant mice that were verified to demonstrate no perlecan expression via northern blot and immunoprecipitation (Arikawa-Hirasawa et al., 1999). These models also displayed embryonic death, chondrodysplasia, and skeletal abnormalities (Arikawa-Hirasawa et al., 1999). Perlecan animal models and subsequent biologic effects are depicted in Figure 1.

Xu et al. (2016) first utilized tissue-dependent conditional animal models of perlecan via the creation of knockout mice that lacked perlecan in skeletal muscle, yet restored perlecan expression in cartilage via a perinatal-lethality rescue mechanism involving the *Col2a1* promoter and enhancer, which successfully allowed tissue-dependent roles of perlecan to be explored. This model has since been used to study perlecan’s specific role in neurogenesis, (Kerever et al., 2014), vascularization of bone (Ishijima et al., 2012), and maintenance of the blood brain barrier (Nakamura et al., 2019).

### 3.2 Perlecan in cartilaginous development

Perlecan has been established as a proteoglycan critical for cartilaginous and chondrocyte formation in the developing fetus (Arikawa-Hirasawa et al., 1999; Costell et al., 1999). Perlecan surrounds stem cell niches in rudimentary cartilage, as seen in Table 1 ^22^.There, the molecule acts as a signal for FGF-2 and FGF-18 induced migration and coordination of chondroprogenitor stem cells necessary to effectively cavitate rudimentary joints. FGF-2 allows mesenchymal stem cells to adopt the chondrogenic phenotype (Shu et al., 2016). Furthermore, the differentiation and hypertrophy of such chondrocytes allows ossification centers to form via FGF-18 induced osteogenesis. Once mineralization occurs, Perlecan is critical in establishing homeostasis and maintenance of permanent cartilage by regulating FGF-2 and FGF-18 growth factor bioavailability (Shu et al., 2016). This allows cartilage to slowly renew while maintaining the structural integrity given to it by FGF-18 induced structurally sound columnar chondrocyte arrangements within the growth plate. Additionally, *HSPG-2* knockout mice were found to have defective endochondral ossification with disorganized collagen fibrils (Arikawa-Hirasawa et al., 1999) and chondrocyte columnar structures. Additionally, perlecan-null mice demonstrated decreased chondrocyte proliferation and dyssegmental ossification of the spine (Arikawa-Hirasawa et al., 2001). Two diseases arise from frameshift mutations within the *HSPG-2* gene, including the heterozygous and milder chondrodysplasia, Schwartz-Jampel Syndrome, and the homozygous neonatal lethal disorder, Silverman-Handmaker Dyssegmental Dysplasia. The pathology of both diseases is contributed to by a functional diminishment or absence of perlecan and thus unstable extracellular matrix support in development (Arikawa-Hirasawa et al., 1999; Arikawa-Hirasawa et al., 2001).

**TABLE 1:** Perlecan’s Interaction with FGF-2 and FGF-18 in Tissue Development. A summary of the relationship between specific growth factors and perlecan in relation to development of various cartilaginous and neurologic tissue.

Developmental pathway	Perlecan’s signaling mechanism	Effect	Source
**Rudimentary Cartilage**	FGF-2 and FGF-18 mediated migration and proliferation of chondroprogenitor stem cells into chondrocytes and osteocytes	Coordinated cavitation of joints and formation of rudimentary cartilage	Hayes et al. (2022)
**Permanent Cartilage**	Homeostasis and maintenance via regulation of FGF-2 and FGF-18 bioavailability	Balance between self-renewal and continuous structural support via columnar organization of chondrocytes	Hayes et al. (2022)
			Shu et al. (2016)
**Hippocampal Neural Tissue**	HS side chain acts as a co-receptor for FGF-2 in dentate gyrus, affecting chemokines and growth factors within neural stem cell niches	Differentiation into pluripotent cells critical for neural pathways in developing brain	Melrose (2022)

### 3.3 Perlecan and pathophysiology of musculoskeletal disease

**FIGURE 2:**
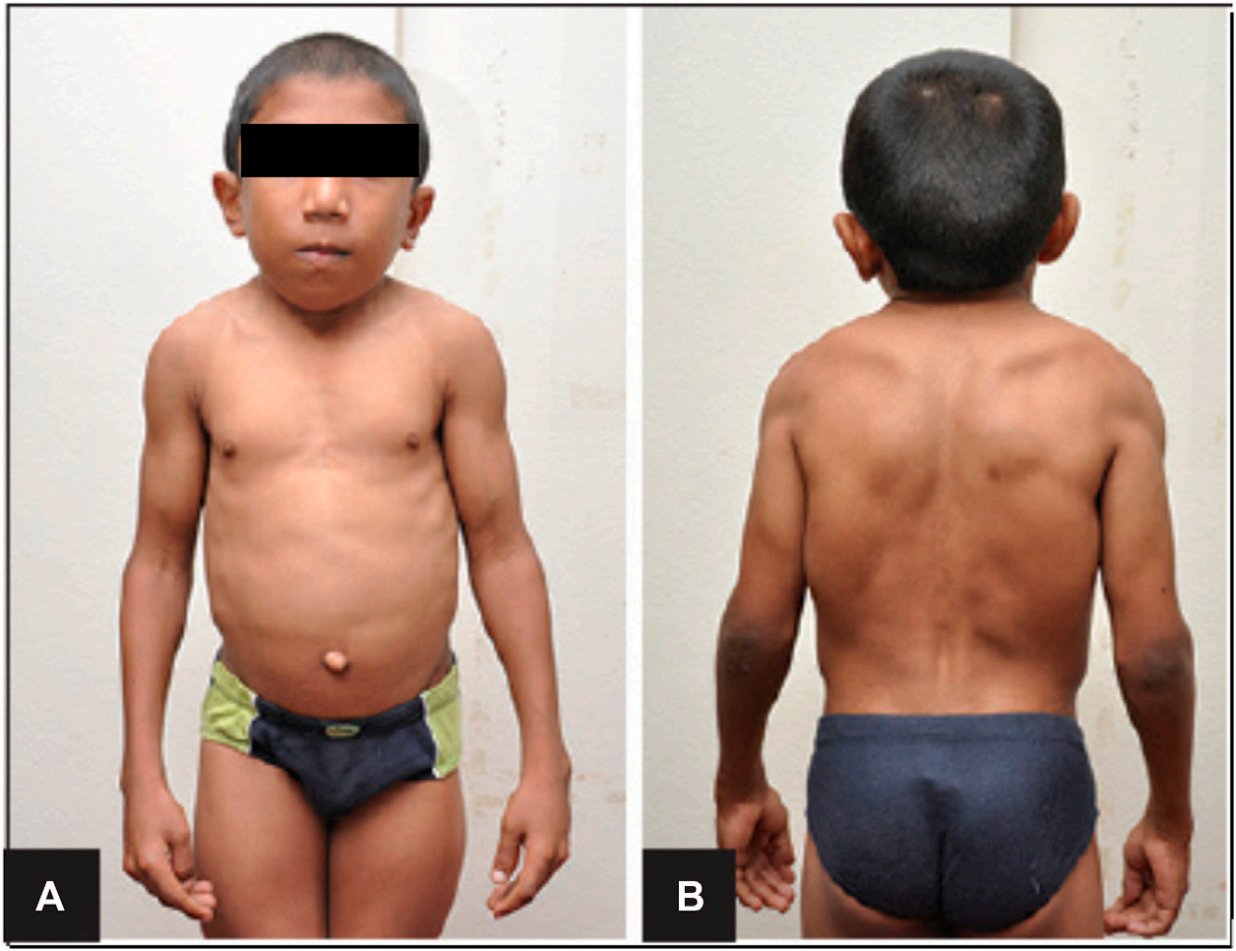
Clinical Features of Schwartz-Jampel Syndrome. Depicted are facial dimorphism, pigeon breast, myotonia. **(A)**. Shows an anterior view with prominent features of misshapen stature. **(B)**. Shows a posterior view with prominent myotonia. Copied with permission from Professor Shelley Bhaskara, Founding Editor in Chief, Archives Medicine Health Sciences (Roger et al., 2012).

Schwartz-Jampel Syndrome is an autosomal recessive disorder with one affected allele of the *HSPG-2* gene that results in less than 10% of functional perlecan levels in the skeletal muscle, fibroblasts, heart and the kidney (Martinez et al., 2018). This disorder is characterized by growth plate disorganization and failed union of the chondro-osseous junction, resulting in features such as misshapen and shortened long bones, facial dimorphism, pigeon breast and myotonia (Costell et al., 1999; Stum et al., 2008; Martinez et al., 2018). Such clinical symptoms of patients with this disorder is depicted in Figure 2. Patients of this disorder typically survive without any alterations to life expectancy; however, the severity of disease is inversely correlated with the amount of functional perlecan secreted into the extracellular matrix in development (Stum et al., 2008).

Silverman-Handmaker Dyssegmental Dysplasia (DDSH) is the more severe form of the two perlecan associated chondrodysplasia disorders. DDSH arises from a frameshift mutation in both alleles of the *HSPG-2* gene, resulting in premature termination and a truncated perlecan protein that undergoes proteolytic degradation (Arikawa-Hirasawa et al., 2001; Martinez et al., 2018). This results in a functionally null gene and absent perlecan secretion into the extracellular matrix, rendering outcomes of affected neonates as lethal. This autosomal recessive chondrodysplasia is characterized by anisospondyly, micromelia, flat facial features, encephalocele, and dysplastic ossification of the spine (Arikawa-Hirasawa et al., 2001). The complete lack of perlecan in this disorder also results in shortened growth plates, dyssegmental organization of growing bones, and degeneration of cartilage, likely from unstable extracellular matrix scaffolding required for cell organization (Arikawa-Hirasawa et al., 1999; Costell et al., 1999; Arikawa-Hirasawa et al., 2001).

In addition to diseases arising from diminished perlecan levels, the pathophysiology of sarcopenia and OA seem to involve increased, rather than decreased, perlecan expression. Sarcopenia is the process of age-related skeletal muscle atrophy and was determined to arise due to satellite cell apoptosis and erroneous capillary function (Wang et al., 2014). Due to perlecan and its C terminal fragment, endorepellin, acting via VEGFR2 on endothelial cells, Bechet et al. examined if either molecule was upregulated in sarcopenic models. Utilizing mouse gastrocnemius, it was found that there was increased apoptosis of capillary endothelial cells, expression of endorepellin, and ECM fibrosis (Wang et al., 2014) in aged specimens compared to controls. Endorepellin functions in an inhibitory manner on receptors crucial for angiogenesis, such as VEGFR1 and 2, and therefore may negatively impact skeletal muscle capillary health in aging individuals (Goyal et al., 2011; Wang et al., 2014; Douglass et al., 2015).

**FIGURE 3:**
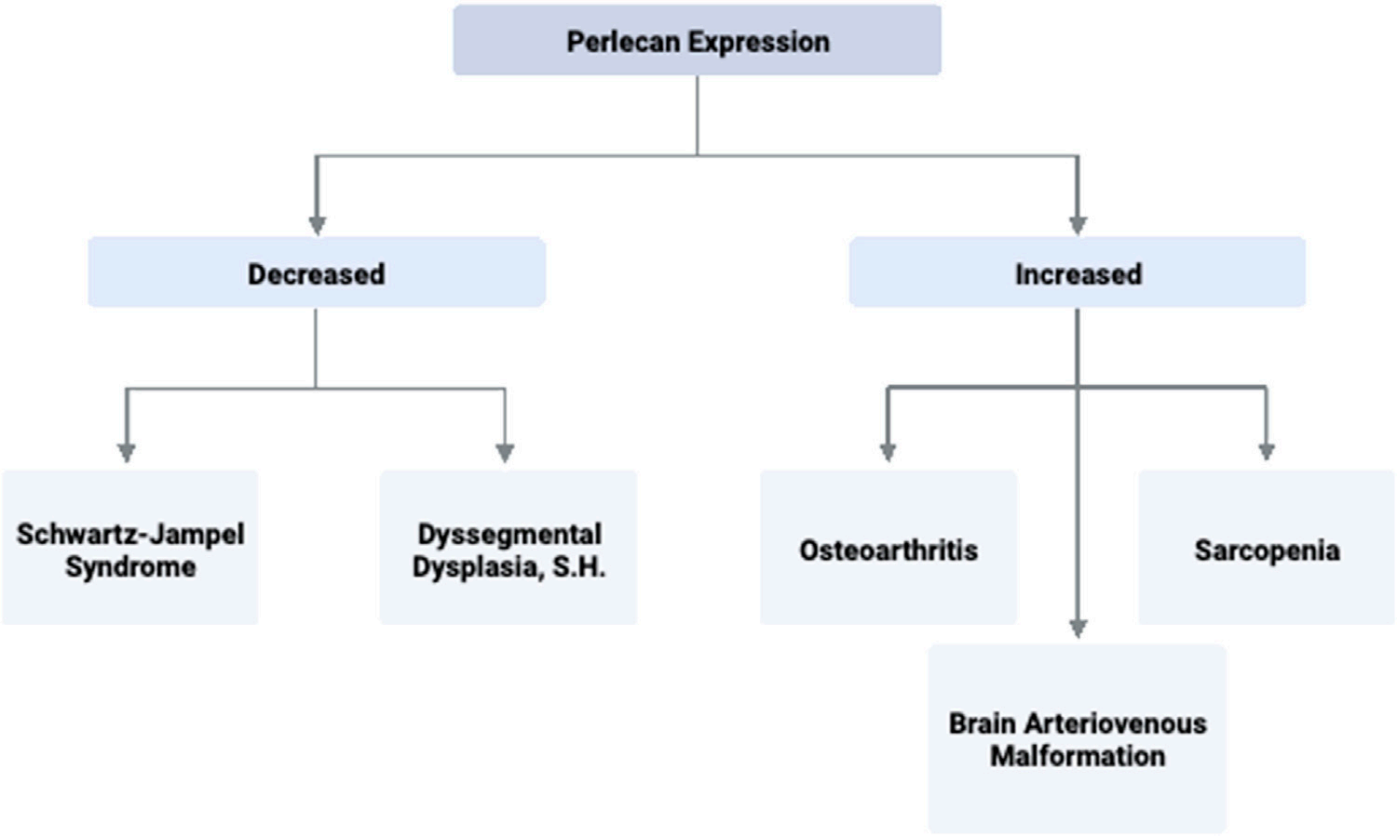
Perlecan Expression and Associated Disease States. Perlecan expression levels are implicated in several significant pathologies due to its ability to promote or inhibit processes such as angiogenesis, extracellular matrix stability, and capillary health. Created with BioRender.com.

Perlecan is also found to be implicated in the pathophysiology of OA. OA affects approximately 27 million US adults and is characterized by deterioration of cartilage, synovitis, and osteophyte formation (National Collaborating Centre for Chronic Conditions, 2008; Kaneko et al., 2013). Such pathology results in stiffness and pain of the joints, making OA one of the leading causes of mobility impairment in elderly populations (National Collaborating Centre for Chronic Conditions, 2008). The work of Arikawa-Hirasawa et al. using perinatal lethality rescued perlecan-knockout mice (*HSPG-2* ^−/−^) examined perlecan’s role in forming osteophytes. In this model, perlecan is lacking in all synovial fluid and joint tissue besides cartilage. Arikawa-Hirasawa et al. utilized both surgical and TGF-B induced osteophyte formation methods to examine osteophyte characteristics. In both models, *HSPG-2* knock-out mice had significantly reduced osteophyte size and maturation (Kaneko et al., 2013). This expands on the finding that primary synovial cells upregulate TGF-B and perlecan in patients with OA (Dodge et al., 1995). Thus, synovial perlecan may play an important role in osteophyte development and inhibition of such signaling pathways may serve as an effective target for OA therapeutics. Perlecan’s involvement and mechanism of action in the musculoskeletal diseases discussed is summarized in Table 2 and Figure 3.

**TABLE 2:** Perlecan’s Involvement in the Pathophysiology of Various Musculoskeletal Disease States. Altered levels of perlecan expression exhibit an important role in the development of systemic musculoskeletal disorders. Created with BioRender.com.

Musculoskeletal disease	Clinical features	Perlecan levels	Mechanism
**Silverman-Handmaker Dyssegmental Dysplasia**	Anisospondyly, micromelia, encephalocele	Absent	Homozygous mutation in HSPG-2 - > absent endochondral ossification
**Schwartz-Jampel Syndrome**	Facial dimorphism, pigeon breast and myotonia	Diminished	Heterozygous mutation in HSPG-2 - > growth plate disorganization and cartilage degeneration
**Sarcopenia**	Age related skeletal muscle atrophy	Diminished	Increased endorepellin:perlecan ratio - > apoptosis of capillary endothelial cells in skeletal muscle
**Osteoarthritis**	Osteophyte formation, Inflammation, pain	Elevated	Perlecan signals upregulation of TGF-β - > pathological cell proliferation and chondrogenesis
**Brain Arteriovenous Malformation**	Osteophyte formation, Inflammation, pain	Elevated	Domain V upregulates VEGF and TGF-β- > increased angiogenesis and nibus formation

### 3.4 Perlecan in neural development

Just as perlecan is necessary for osseous and cartilaginous function, it is also critical in the development and pathophysiology of neural systems. Perlecan’s side chains interact with FGF-2 within the neural stem cell niches of the sub-ventricular and sub-granular dentate gyrus of the hippocampus, effectively promoting stem cell self-renewal and neural propagation (Table 1) (Hayes et al., 2022). Furthermore, reduction of Perlecan’s signal transduction structure with FGF-2 in the sub-ventricular zone has been seen in aged models (Hayes et al., 2022). Such effect is thought to occur by diminished cell survival and proliferation via failure of FGF-2 induced phosphorylation of extracellular signal-regulated kinase (Erk1/2). Perlecan is also critical for proper cephalic development, as *HSPG-2* ^*−/−*^ knockout mice experienced diminished development of the hindbrain and forebrain with death or exencephaly occurring shortly after fertilization (Arikawa-Hirasawa et al., 1999). Additionally, perlecan null mouse models experience collapsed brain vesicles, likely due to weakened basement membranes that cannot withstand the shear stress of developing neural structures of which perlecan can provide structural integrity (Arikawa-Hirasawa et al., 1999; Costell et al., 1999; Martinez et al., 2018). As such, functionally null models without perlecan are embryonic lethal due to weakened capillary walls and subsequent bleeding in the brain, lungs, and heart of the developing embryo (Martinez et al., 2018).

### 3.5 Perlecan: therapeutic potential and pathophysiology of neurological disease

Due to its central role in neurodevelopment and angiogenesis, perlecan has been examined as a therapeutic for ischemic stroke. Stroke is the primary contributor to long-term disability in the United States (Roger et al., 2012). In addition, the only viable treatment remains tissue plasminogen activator (tPA), though this requires an incredibly narrow efficacious window of only 4.5 h post ischemic attack. Our recent work investigated the effects of domain V of perlecan as an effective broad therapy for ischemic stroke agent from previous findings that perlecan may be neuroprotective and that domain V is upregulated in the post-stroke human brain (Lee et al., 2011). We found that in stroke mouse models receiving domain V 7 days after ischemic event, peri-infarct excitatory synapse generation extended further into the neocortex compared to mice that did not receive domain V. Furthermore, perlecan deficient mice demonstrated less neuroblast precursor cells post-stroke than those with functional perlecan expression (Trout et al., 2021). Such neuro-reparative outcomes may function through the α2β1 integrin receptor signaling pathway, as blockade of this receptor mostly ameliorated the observed effects, although perlecan can bind to several other integrin receptors. Lastly, compared to control mice, mice who received perlecan domain V also experienced better neurological outcomes post ischemic stroke via regulation of pericyte migration. Mediating pericyte transport and facilitating connection to the extracellular matrix is critical for repair of the blood brain barrier that becomes “leaky” after ischemia (Roberts et al., 2012). Domain V administration recruited pericytes to the BBB via a PDGFR-β and integrin α5β1 signaling mechanism, and acted to regulate focal adhesion of extracellular matrix components and stabilized the actin cytoskeleton (Nakamura et al., 2019). Perlecan knock-out mice had a lower overall survival rate, along with increased weight loss and neuro-deficits compared to control mice (Nakamura et al., 2019). Perlecan’s role in restoring the blood brain barrier post stroke via pericyte modulation demonstrates its potential as an efficacious therapeutic. As domain V administration additionally enhanced synaptic neurogenesis and neural precursor cells in this model, this makes perlecan and its derivatives a promising potential therapy that warrants further investigation.

Altered levels of perlecan has also been associated with the pathophysiology of brain arteriovenous malformations (BAVM). BAVM is a grouping or “nidus” of dysplastic arteries and veins in the brain that lack a true capillary bed (Kim et al., 2009). Subsequently, they serve as a shunt that sends arterial blood into venous circulation, putting the individual at risk of intracranial hemorrhage and mortality (Kim et al., 2009). BAVMs are overly angiogenic via increased *VEGF* signaling, a pathway through which perlecan domain V participates in. In human BAVM specimens, perlecan’s endogenously cleaved segment, domain V, and its pro-angiogenic signaling molecule, α5β1 integrin, were both increased compared to control brain tissue (Kahle et al., 2012). Domain V levels were 14 times higher in the BAVM specimens, and VEGF and TGFβ were increased 9-fold compared to control (Kahle et al., 2012). This provides evidence that perlecan domain V’s upregulation of VEGF may be critical for formation of a BAVM nidus and that inhibition of this mechanism should be investigated further as a therapeutic target.

**FIGURE 4:**
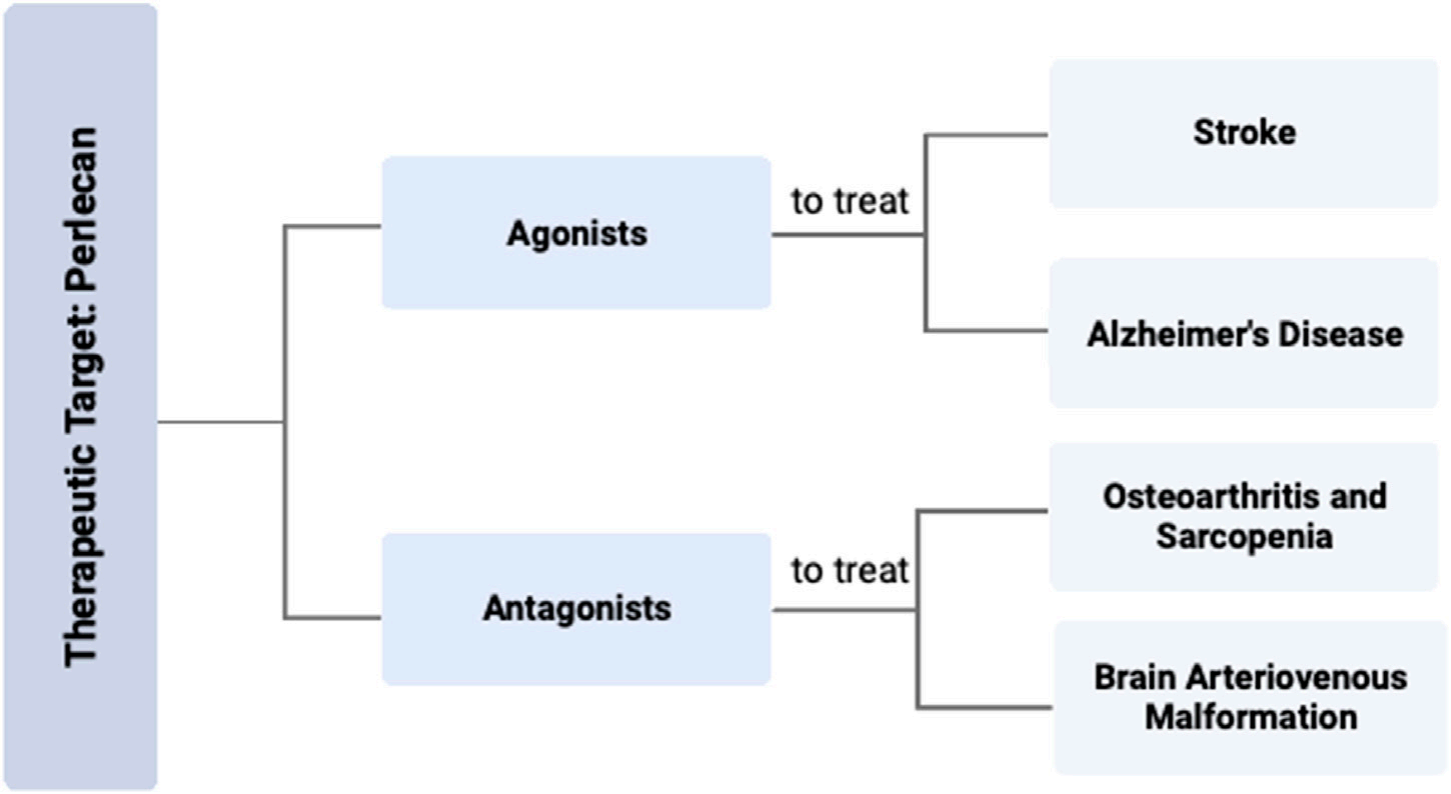
Perlecan Modification as Therapeutic Target for Various Diseases. Targeting the effects of perlecan with either an agonist or antagonist could be an effective strategy for the treatment of various disease states. Specific therapy with a perlecan agonist can be used to treat stroke and AD, while a perlecan antagonist can be used for OA, sarcopenia, or BAVMs. Created with BioRender.com.

Perlecan’s angiogenic proclivity may also cause it to be a potential therapeutic target for the treatment of AD. AD remains a leading cause of disability and mortality in the United States, with resultant deaths increasing by 146% from 2000 to 2018 (Alzeimer’s Association, 2020). The disease is characterized by neurofibrillary tangles and misfolded deposits of amyloid-β (Aβ) of various sizes forming in the cerebrovasculature. This consequently results in damaged endothelial lining, decreased VEGF levels and angiogenesis, and overall diminished neurovascular status that promotes the hallmark symptoms of depression, confusion, loss of memory, and dementia (Parham et al., 2014; Alzeimer’s Association, 2020). Endothelial damage subsequently disrupts the blood brain barrier and therefore diminishes Aβ clearance pathways, resulting in neurotoxic buildups of Aβ (Parham et al., 2014; Nakamura et al., 2019). Experimental administration of domain V to AD mouse models effectively blocked the reduced cerebral endothelial cell proliferation that resulted from Aβ_25-35_ administration (Parham et al., 2014). Administering perlecan domain V also restored the ability of mouse brain endothelial cells to begin forming “tube” like capillary structures even with the presence of Aβ_25-35._ Such findings suggest perlecan domain V may outcompete the Aβ plaques for *VEGFR2*, and thus may restore angiogenesis and endothelial cell integrity to more functional and healthy levels. As domain V of perlecan is known to be pro-angiogenic through α5β1 integrin signaling and upregulation of *VEGFR2*, this may ultimately be the mechanism underlying its anti-angiogenic effects to Aβ and the way in which it provides protection to the cerebrovasculature (Aviezer et al., 1994). Experimental treatments in animal models using perlecan level manipulation are summarized in Table 3 and Figure 4.

**TABLE 3:** Altering Perlecan levels as Potential Therapeutic Treatment for Ischemic Stroke, Alzheimer’s Disease, and Osteoarthritis. A summary of neurological and musculoskeletal disease states in experimental models is shown. Treatment with DV administration or alterations in perlecan expression in synovium ameliorates deficits seen in neuropathological insults and osteoarthritis. Created with BioRender.com.

Disease	Perlecan levels	Experimental treatment	Outcome	Mechanism
**Ischemic Stroke**	Domain V Upregulated Post TIA	Domain V administration	Increased neuroprotection, neurogenesis, and recovery	a2 (31 integrin receptor stimulation, enhanced pericyte migration
**Alzheimer’s Disease**	Unknown	Domain V administration	Increased endothelial cell healing	Increased angiogenesis via VEGF/VEGFR2 pathway
**Osteoarthritis**	Elevated	HSPG-2 knock-out mice lacking all perlecan in synovium	Reduced osteophyte formation, cell proliferation	Upregulation of TGFB signaling pathways in synovium

## 4 Discussion and future directions

Overall, perlecan is an essential proteoglycan within the basement membrane which provides critical function for neural and cartilaginous development that can result in significant disease states when produced in dysregulated quantities. From this review, increased perlecan concentration was associated with sarcopenia, OA, and BAVMs, as well as being produced in greater quantities in animal models of stroke. Perlecan levels were found to be decreased in the chondrodysplasia syndrome known as SJS, and absent in the lethal DDSH.

For disorders with increased perlecan, therapeutic blockade of the effects of perlecan signaling may provide reduction in associated symptoms and disease. As perlecan binds to both the α5β1 integrin and VEGFR2 receptor, tissue specific inhibitors of these proteins may therefore yield the most beneficial effect in therapeutic targeting for treating the pathophysiology of sarcopenia, OA, and BAVM. For disorders with decreased perlecan, administration of purified/recombinant perlecan or its derivatives may provide therapeutic relief. Although such disorders arise from genetic mutations in the *HSPG-2* gene, there have been no studies which examine if exogenously administered perlecan to SJS patients would provide any benefit. Due to the genetic nature of these musculoskeletal diseases, targeting the *HSPG-2* gene via CRISPR technology may restore the necessary perlecan levels needed for proper musculoskeletal health.

Animal models suggest that administration of perlecan may be beneficial as a broad spectrum therapeutic for stroke victims, as our group has shown it to be neuroprotective, promote neurogenesis, and stabilize the blood brain barrier in our studies. As there is currently such limited therapeutics for immediate ischemic stroke, further investigation on the broad therapeutic potential of domain V is warranted and encouraged. Lastly, perlecan domain V has been shown to protect against the neurotoxic effects of Aβ-amyloid deposits in AD models. As both stroke and AD are both extremely prevalent causes of neurological and cognitive deficits, future work into the potential of perlecan domain V as a therapeutic index is imperative.

This review serves as the first to summarize neurological and musculoskeletal diseases in the context of varied perlecan expression and to discuss its potential as a therapeutic index to ameliorate disease pathophysiology. Future work examining the exact role of perlecan and its derivatives in these disorders may reveal new pharmacological therapeutics and thus contribute overall to reduced suffering in these diseases.
